# Endobronchial Lipoma Treated With Endoscopic Microwave Coagulation to Relieve Atelectasis: The First Case Report

**DOI:** 10.1002/rcr2.70313

**Published:** 2025-08-27

**Authors:** Eri Takase, Atsushi Washioka, Keita Nakaguchi, Mitsumasa Kawago, Fumihiro Tanaka, Masanori Kitaichi

**Affiliations:** ^1^ Department of Respiratory Medicine National Hospital Organization Minami Wakayama Medical Center Tanabe, Wakayama Japan; ^2^ Internal Medicine III Wakayama Medical University Wakayama Japan; ^3^ Department of Thoracic Surgery National Hospital Organization Minami Wakayama Medical Center Tanabe, Wakayama Japan; ^4^ Department of Radiology National Hospital Organization Minami Wakayama Medical Center Tanabe, Wakayama Japan; ^5^ Department of Pathology National Hospital Organization Minami Wakayama Medical Center Tanabe, Wakayama Japan

**Keywords:** atelectasis, endobronchial lipoma, endobronchial therapy, high perioperative risk, microwave coagulation

## Abstract

Various endobronchial therapies, as well as complete surgical resection, have recently been reported. Among them, microwave coagulation is a promising endobronchial therapy for a wide range of neoplasms due to its high efficacy and safety. Meanwhile, endobronchial lipoma is a benign tumour that is rarely observed in the lungs. To our knowledge, the efficacy of microwave coagulation has not been reported in the treatment of endobronchial lipoma. Here, we report the effectiveness of endoscopic microwave coagulation therapy in safely resolving atelectasis and cough caused by endobronchial lipoma. Surgical resection was deemed to be unsuitable in this case due to the likelihood of extensive surgery such as right lower lobectomy being required, and comorbidities including poorly controlled diabetes mellitus. There was an elevated risk of perioperative complications. This report presents a new, effective, and minimally invasive treatment option for endobronchial lipoma in endoscopic microwave coagulation to relieve atelectasis.

## Graphical abstract

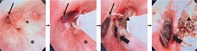

## 1 Introduction

Endobronchial lipoma is a rare benign tumour, accounting for approximately 0.01% of all bronchial tumours [1]. It predominately occurs in men aged 50–80 years, and recognised risk factors include smoking and obesity. Although benign, it may cause respiratory symptoms such as cough or dyspnea. Therapeutic intervention becomes necessary with the development of complications such as pneumonia or atelectasis. The advancement of endobronchial therapies now enables effective management of endobronchial tumours, particularly in patients that would be considered to be poor surgical candidates. Microwave coagulation has emerged as a promising therapy due to its uniform thermal delivery and minimal tissue carbonisation. However, its use for endobronchial lipoma has not been previously described. We present a case of symptomatic endobronchial lipoma that was successfully treated with endoscopic microwave coagulation therapy.

## 2 Case Report

**FIGURE 1:**
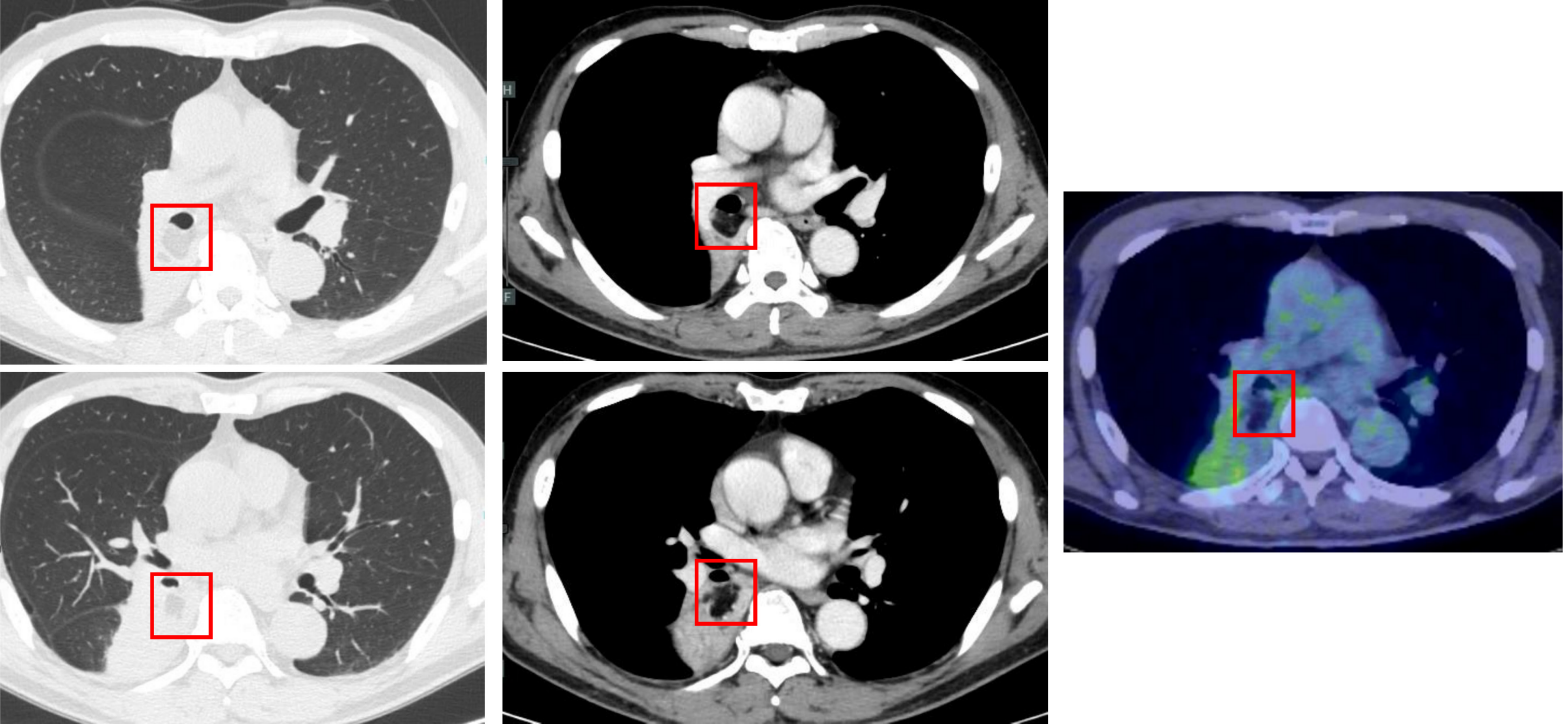
Chest computed tomography and positron emission tomography‐computed tomography at first visit.

**FIGURE 2:**
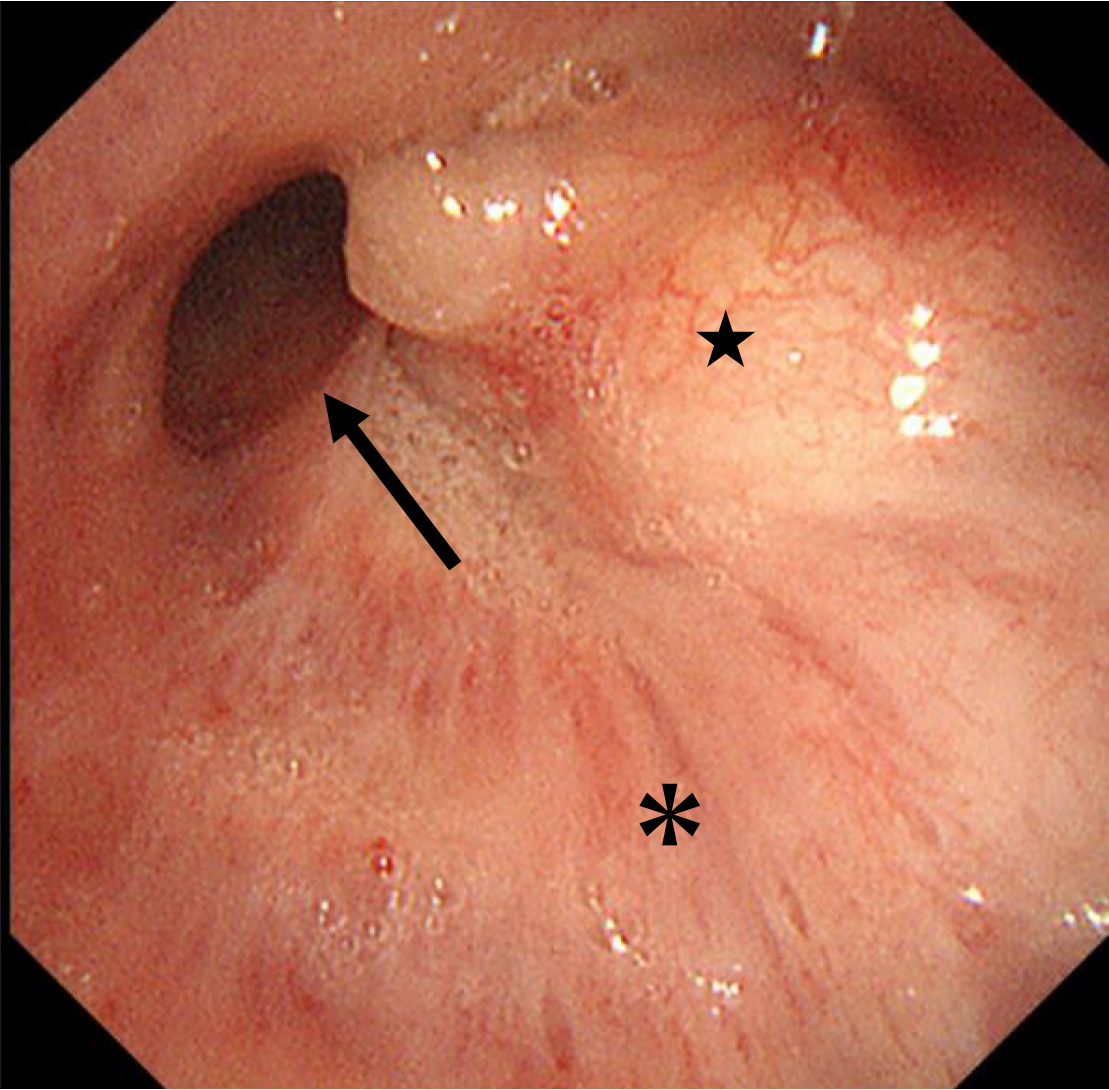
Bronchoscopy revealed a mass extending from the truncus intermedius (*) to the lower lobe bronchus (★). Thin arrow shows middle lobe bronchus.

**FIGURE 3:**
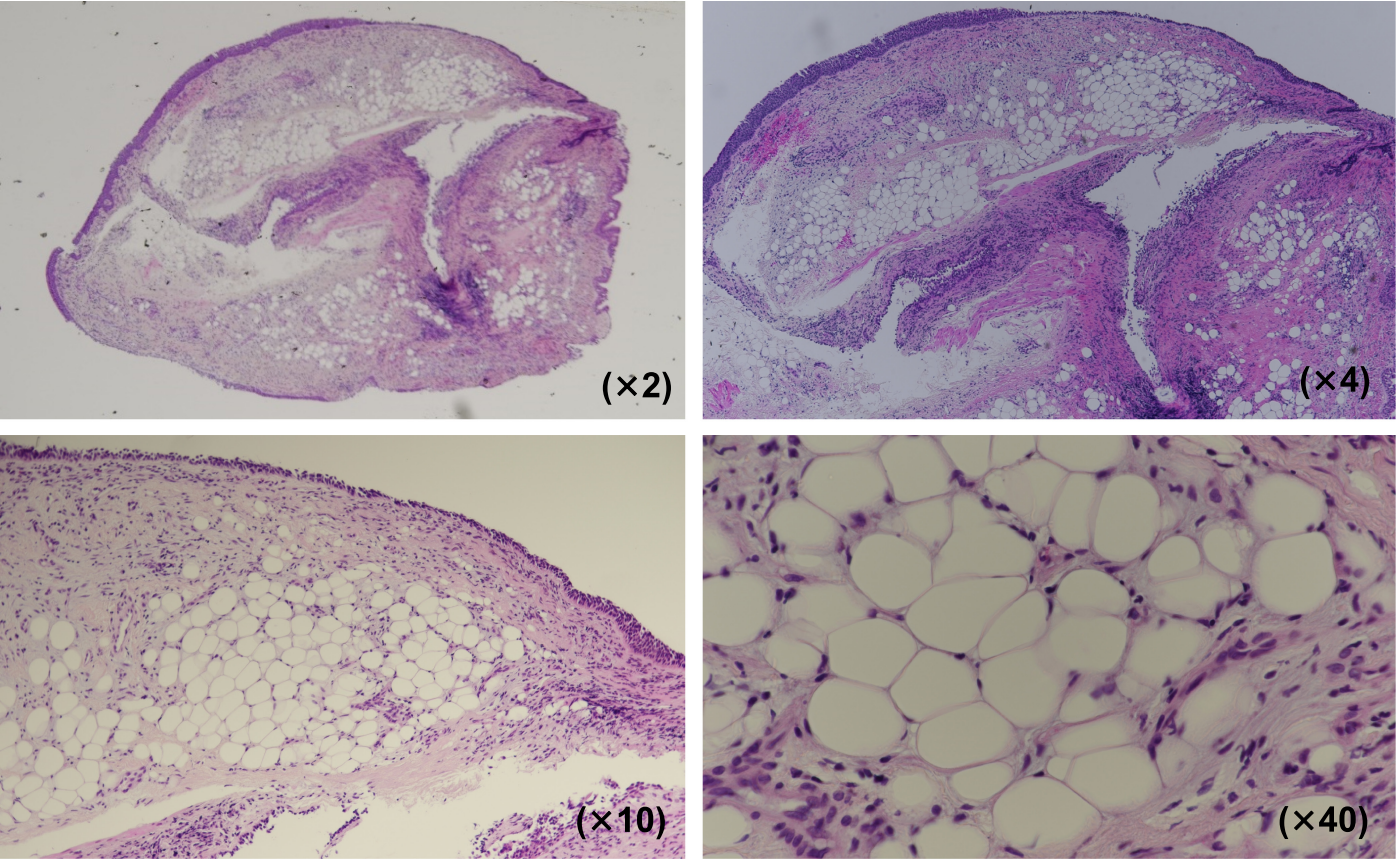
Pathological imaging (Haematoxylin–eosin staining).

**FIGURE 4:**
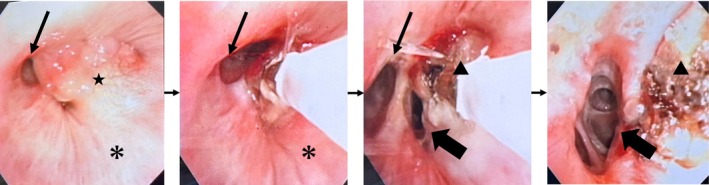
A mass extended from the truncus intermedius (*) to the lower lobe bronchus (★). After tumour debulking via endoscopic microwave coagulation therapy, the lumens of the middle lobe (thin arrow), medial basal segmental bronchus (thick arrow), and right B6 (▲) were fully visualised.

**FIGURE 5:**
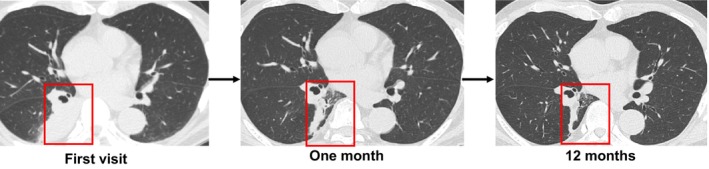
Time course of the chest imaging.

A 72‐year‐old man presented with a persistent cough lasting 1 month. Chest computed tomography revealed an oval tumour (25 × 17 mm) with a CT attenuation of −82 Hounsfield Units (HU) in the right B6 bronchus, resulting in atelectasis of the superior segment (Figure 1). Positron emission tomography‐computed tomography showed no fluorodeoxyglucose uptake, suggesting the absence of malignancy. Bronchoscopy revealed a yellowish, smooth‐surfaced mass extending from the truncus intermedius to the lower lobe bronchus that was obstructing the right B6 (Figure 2). Histopathology showed mature adipocytes covered by respiratory epithelium, which is consistent with endobronchial lipoma (Figure 3). Surgical resection was considered, but complete removal would likely have required a right lower lobectomy due to the tumour's extension beyond the bronchial wall. Given this extent of surgery being required and the patient's poorly controlled diabetes mellitus (HbA1c 8.7%), the multidisciplinary team judged that surgical intervention would carry an elevated risk of perioperative complications, particularly infection. Endobronchial therapy was therefore recommended as a safer and less invasive alternative. After tumour debulking via endoscopic microwave coagulation therapy, the lumens of the middle lobe, medial basal segmental bronchus, and right B6 were fully visualised (Figure 4). Atelectasis resolved, and the patient's respiratory symptoms improved. Twelve months after the procedure, follow‐up imaging showed no evidence of tumour recurrence (Figure 5).

## 3 Discussion

Surgical resection has traditionally been performed for endobronchial lipomas, particularly in cases with diagnostic uncertainty, extensive peripheral lung damage, extra‐bronchial growth, sub‐pleural components, or when there were technical limitations related to bronchoscopic access [2]. However, as with other airway tumours, surgery has associated risks, such as postoperative infection or respiratory impairment. Endobronchial therapies are gaining favour due to their minimally invasive nature and repeatability. Recent literature documents the successful use of rigid bronchoscopy, high‐frequency snare, Nd‐YAG laser, and cryotherapy in the management of endobronchial lipomas [1, 3]. The availability of these modalities varies by institution, and expanding the armamentarium of therapeutic options is thought to be beneficial. To our knowledge, this is the first report of the use of endoscopic microwave coagulation therapy for endobronchial lipoma. Microwave coagulation utilises dielectric hysteresis to generate uniform heat, resulting in efficient tissue ablation. Compared with other techniques, such as laser therapy or electrocautery, microwave therapy offers deeper, more uniform thermal penetration with minimal carbonization. It has been shown to be effective in high‐impedance tissues such as the lungs and is less influenced by heat‐sink effects [4]. Microwave therapy has been demonstrated to be safe in pulmonary applications, with a reported device‐related adverse event rate of only 3.3%, mainly mild hemoptysis [5]. In patients for whom surgery is contraindicated due to comorbidities or high perioperative risk, endoscopic microwave coagulation provides a viable alternative. This case highlights the utility, safety, and potential advantages of microwave coagulation therapy for the bronchoscopic management of endobronchial lipoma.

## Author Contributions

**Eri Takase:** writing – original draft, investigation, data preparation, supervision, writing – review and editing, conceptualisation. **Atsushi Washioka**, **Keita Nakaguchi**, **Mitsumasa Kawago**, **Fumihiro Tanaka**, and **Masanori Kitaichi:** investigation, data preparation.

## Consent

The authors declare that written informed consent was obtained from the patient for publication of this case report and accompanying images using the consent form provided by the Journal.

## Conflicts of Interest

The authors declare no conflicts of interest.

## Data Availability

Data sharing is not applicable to this article as no new data were created or analyzed in this study.
