# Correction to “Endobronchial Lipoma Treated With Endoscopic Microwave Coagulation to Relieve Atelectasis: The First Case Report”

**DOI:** 10.1002/rcr2.70350

**Published:** 2025-09-22

E. Takase, A. Washioka, K. Nakaguchi, M. Kawago, F. Tanaka, and M. Kitaichi, “Endobronchial Lipoma Treated With Endoscopic Microwave Coagulation to Relieve Atelectasis: The First Case Report,” *Respirology Case Reports* 13, no. 8 (2025): e70313, https://doi.org/10.1002/rcr2.70313.

The following errors were identified:

1. In the Introduction section, the sentence ‘Endobronchial lipoma is a rare benign tumour, accounting for approximately 0.01% of all bronchial tumours [1]’. is incorrect. It should be corrected to:
  ‘Endobronchial lipoma is a rare benign tumour, accounting for approximately 0.1% of all bronchial tumours [1]’.
2. Reference [1] is incorrect and it should be corrected to:
  H. Shah, L. Garbe, E. Nussbaum, J. F. Dumon, P. L. Chiodera, and S. Cavaliere, “Benign Tumors of the Tracheobronchial Tree. Endoscopic Characteristics and Role of Laser Resection,” *Chest* 107, no. 6 (1995): 1744–1751, https://doi.org/10.1378/chest.107.6.1744.
3. In the Discussion section, reference citation 1 should be deleted from the sentence ‘Endobronchial therapies are gaining favour due to their minimally invasive nature and repeatability. Recent literature documents the successful use of rigid bronchoscopy, high‐frequency snare, Nd‐YAG laser, and cryotherapy in the management of endobronchial lipomas [1, 3]’. It should be corrected to:
  ‘Endobronchial therapies are gaining favour due to their minimally invasive nature and repeatability. Recent literature documents the successful use of rigid bronchoscopy, high‐frequency snare, Nd‐YAG laser, and cryotherapy in the management of endobronchial lipomas [3]’.

We apologise for these errors.

